# Expression and significance of serum vitamin D and LL-37 levels in infants with bacterial pneumonia

**DOI:** 10.3389/fped.2022.989526

**Published:** 2022-11-09

**Authors:** Shaojie Zhao, Yixiu He, Menglan Pan, Bingzhang Chen, Siqi Zhang, Yufeng Zhang, Yafei Zhu

**Affiliations:** ^1^ Department of Pediatrics, The Affiliated Hospital of Hangzhou Normal University, Hangzhou, China; ^2^ Department of Pediatrics, Yuyao People's Hospital of Zhejiang Province, Yuyao, China

**Keywords:** vitamin D, antimicrobial peptide, LL-37, bacterial pneumonia, infants

## Abstract

**Objective:**

To investigate the significance of vitamin D and human antimicrobial peptide LL-37 in the occurrence and development of bacterial pneumonia in infants.

**Methods:**

From January 2021 to January 2022, 80 infants with bacterial pneumonia were selected, including 33 cases of gram-positive bacterial infection (GP) and 47 cases of gram-negative bacterial infection (GN). During the same period, 40 infants who underwent health examination in The Affiliated Hospital of Hangzhou Normal University served as the healthy control group. On the day of admission, peripheral blood was collected from pneumonia patients, and during physical examination of controls; and serum LL-37 levels were measured by enzyme-linked immunosorbent assay (ELISA) and serum *25-hydroxyvitamin D [*25(OH)D] levels were measured by electrochemiluminescence. The differences in serum LL-37 and 25(OH)D levels and their correlation with disease severity were compared. Pearson correlation was used to analyze the correlation between serum 25(OH)D and LL-37 levels in infants with bacterial pneumonia.

**Results:**

The levels of 25(OH)D and 25(OH)D deficiency were significantly lower in patients than in controls (all *P* < 0.05), and the levels of serum LL-37 were significantly higher in pneumonia patients than in controls (*P* < 0.05). There was no significant difference in serum 25(OH)D and LL-37 levels between cases with GP and GN (all *P* > 0.05). The serum 25(OH)D level was lower in the severe pneumonia group than in the mild pneumonia group and controls, and the 25(OH)D deficiency rate was higher; the difference was statistically significant (all *P* < 0.05). The LL-37 level in the severe pneumonia group was lower than that in the mild pneumonia group but higher than that in the control group, and the difference was statistically significant (*P* < 0.05). The 25(OH)D level was positively correlated with the LL-37 level (*r* = 0.8, *P* < 0.05), and the 25(OH)D level was negatively correlated with procalcitonin (PCT) and length of hospital stay (*r_s_ *= −0.3, −0.3, *P* < 0.05); the LL-37 level was negatively correlated with PCT and length of hospital stay (*r_s_* = −0.4, −0.2, *P* < 0.05) in infants with bacterial pneumonia.

**Conclusion:**

A low level of vitamin D is present in infants with bacterial pneumonia, and its status affects the severity and outcome of pneumonia. The level of LL-37 is increased in infants with bacterial pneumonia, but it shows a downward trend with progression of the disease.

## Introduction

According to the World Health Organization, bacteria are the main pathogens that cause severe pneumonia in children and this disease condition can be life-threatening in severe cases (1). Vitamin D is an important fat-soluble vitamin during the growth process of children. In addition to its regulatory effect on bones and calcium and phosphorus metabolism, it is also involved in the regulation of anti-infection and immune function, as well as in the proliferation, differentiation, and apoptosis of a variety of cells (2). In vitro experiments have confirmed that 25(OH)D enhances the killing ability of immune cells against various types of bacteria (3). 25(OH)D produces antimicrobial peptides, including human antimicrobial peptide LL-37 and human *β*-defensin-2, by activating vitamin D receptors and binding to vitamin D effector element target genes during pathogen infection (4). LL-37 is the only human endogenous antimicrobial peptide in the cathelicidin family that has been discovered so far (5), and it shows a wide range of antibacterial activity against bacteria, viruses, and fungi (6) along with immunomodulatory and chemotactic effects (7, 8), it is named LL - 37 because it contains two lysine residues (L) before the N-terminus, and consists of 37 amino acids (9). The promoter region of LL - 37 gene contains a TAAA sequence, an interleukin 6 nuclear factor - 6 (NF - IL - 6) binding site, and three acute phase response factors (APRF), which can bind to inflammatory factors, thereby increasing LL - 37 gene expression levels (10). 1,25-Dihydroxyvitamin D [1,25(OH)_2_D] is a product modified by CYP27B1-catalyzed 1*α*-hydroxylation of 25(OH)D (11), which can signal through the vitamin D receptor (VDR) and recognize a specific DNA sequence of the vitamin D response element (VDRE) contained in the LL-37 gene to induce the expression of LL-37 (12), while lipopolysaccharide (LPS) induces the expression of LL-37 gene through signals emitted by Toll like receptor (TLR) (13). Currently, few studies have focused on the relationship among vitamin D and LL-37, and bacterial pneumonia in infants. In this study, we aimed to detect the serum levels of 25(OH)D and LL-37 in infants with bacterial pneumonia, and explore their expression and significance in these infants.

## Subjects and methods

### Study subjects

**Figure 1:**
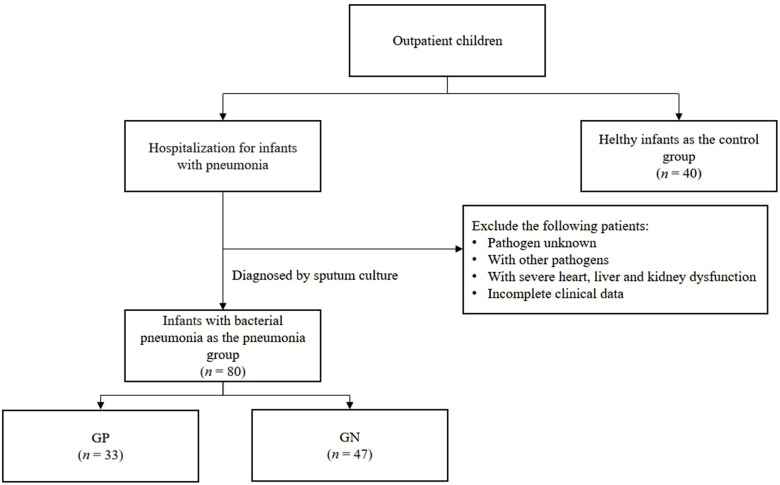
Follow diagram of subject selection (*n*: number).

Eighty infants with bacterial pneumonia diagnosed by sputum culture who were hospitalized in the Department of Pediatrics of The Affiliated Hospital of Hangzhou Normal University from January 2021 to January 2022 were selected. In this study, bacterial pneumonia was diagnosed by comprehensive analysis, including blood routine, CRP, PCT and other peripheral blood infection indicators as well as chest imaging examination, and sputum culture was performed twice on the first and third day of admission in all children to improve the accuracy of etiology. There were 50 males and 30 females with an average age of 10 months [average age of males to females (6:14) (months)]. A total of 40 infants include 23 males and 17 females with an average age of 10 months [average age of males to females (6:16) (months)] were selected as the control group. There were no significant differences in gender and age between the two groups, all *P* > 0.05, which were comparable. In the pneumonia group, there were 33 cases of Gram-positive bacterial infection (GP, 24 cases of Streptococcus pneumoniae infection and 9 cases of Staphylococcus aureus infection) and 47 cases of Gram-negative bacterial infection (GN, 15 cases of Moraxella catarrhalis infection, 14 cases of Haemophilus influenzae infection, 10 cases of Klebsiella pneumoniae infection, and 8 cases of Escherichia coli infections), as shown in Figure 1. According to the diagnostic criteria of the eighth edition of Zhu Futang Practice of Pediatrics (14), there were 23 cases of severe pneumonia and 57 cases of mild pneumonia. All children and guardians of healthy infants gave informed consent and signed the informed consent form. This study was approved by the hospital ethics committee [approval number: 2021 (E2) -KS-025].

#### Inclusion criteria

(1) 28 days < age ≤ 36 months; (2) Fulfillment of the diagnostic criteria for bacterial pneumonia proposed in the Guidelines for the Management of Community-acquired Pneumonia *in Children (2013 Revision*) (15) of the Chinese Pediatric Society; (3) Healthy infants for nearly 30 days without any history of infectious diseases.

#### Exclusion criteria

(1) Age ≤ 28 days, or >36 months; (2) Children with chronic diseases, congenital diseases, or history of long-term use of hormones and immunosuppressive agents; (3) Children with a clear history of respiratory tract, digestive tract, urinary tract, and nervous system infections in the past 30 years; and children with other pathogen infections and children with two or more bacterial infections; (4) Guardians of children refused to participate in this study or had incomplete clinical data.

### Research methods

Two milliliters of peripheral venous blood was collected from all hospitalized children before treatment and centrifuged for 10 min (3,000 r/min, centrifugal radius 70 mm), and the serum was cryopreserved in an ultra-low temperature freezer at −80 °C for future testing. Levels of antimicrobial peptide LL-37 were measured by ELISA, and human antimicrobial peptide ELISA kit was purchased from Shanghai Keshun Biotechnology Co., Ltd. Serum 25(OH)D levels were measured by the Roche electrochemical method, and relevant results were obtained from the clinical laboratory of the hospital, the analyzer is Roche Cobas E602 (Roche Diagnostics Gmbh, Germany, Manufactured 2014).

### Criteria for judging vitamin D levels

vitamin D insufficiency (IS): 25(OH) D < 75 nmo/L; vitamin D sufficiency (S): 75 nmo/L ≤ 25(OH) D ≤ 250 nmo/L (16).

### Statistical methods

SPSS 20.0 software(SPSS Inc., Chicago, IL, United States) was used for collation and analysis. All enumeration data were analyzed by *χ2* test. Measurement data following normal distribution were described as mean ± standard deviation (*x* ± *s*), mean of two samples was compared by two independent samples *t* test, and multiple groups were compared by variance analysis; other measurement data were described as median (interquartile range), and median of two groups was compared by Mann-Whitney *U* test. Correlation analysis was performed using Pearson correlation analysis or Spearman's rank correlation test, and *P* < 0.05 was considered statistically significant.

## Results

### Comparison of serum 25(OH)D and Ll-37 levels

The serum 25(OH)D level was lower in patients than in controls, the 25(OH)D deficiency rate was higher in patients than in controls, and the serum LL-37 level was higher in patients than in controls. The differences were statistically significant (all *P* < 0.05), as shown in Table 1. There was no significant difference in serum 25(OH)D and LL-37 levels between the cases with GP and GN (all *P* > 0.05), as shown in Table 1. The serum 25(OH)D level was the lowest in the severe pneumonia group, and the deficiency rate was the highest in the severe pneumonia group; the difference was statistically significant (*P* < 0.05). The LL-37 level in the severe pneumonia group was lower than that in the mild pneumonia group but higher than that in controls, and the difference was statistically significant (*P* < 0.05), as shown in Table 1.

**Table 1:** Comparison of 25(OH)D and LL-37 levels between groups.

	25 (OH)D (nmol/L)	LL-37 (pg/ml)	25 (OH)D (IS/S)
Patients (*n *= 80)	102 ± 40	317 ± 62	20/60
Controls (*n *= 40)	138 ± 42	239 ± 44	3/37
***t*/*F*/*χ^2^***	−4.4^a^	8.0^a^	5.3^c^
*P*	<0.001	<0.001	0.02
GN (*n *= 33)	103 ± 38	102 ± 42	/
GP (*n *= 47)	327 ± 65	310 ± 59	
***t*/*F*/*χ^2^***	0.1^a^	1.3^a^	
*P*	0.9	0.2	
Severe (*n *= 23)	80 ± 40	296 ± 54	11/12
Mild (*n *= 57)	113 ± 37	329 ± 61	9/48
Controls (*n *= 40)	138 ± 42	239 ± 44	3/37
***t*/*F*/*χ^2^***	16.5^b^	30.1^b^	14.4^c^
*P*	<0.001	<0.001	<0.001

25(OH)D (IS/S): IS/S, insufficiency/sufficiency. GN, Gram-negative bacterial infection group. GP, Gram-positive bacterial infection group.

^a^ Means *t* value. Mean of two samples was compared by two independent samples *t* test.

^b^ Means *F* value. Multiple groups were compared by variance analysis.

^c^ Means ***χ^2^*** value. Enumeration data were analyzed by ***χ^2^*** test.

### Correlation analysis

#### Correlation analysis of serum 25(OH)D and Ll-37 levels

**Figure 2:**
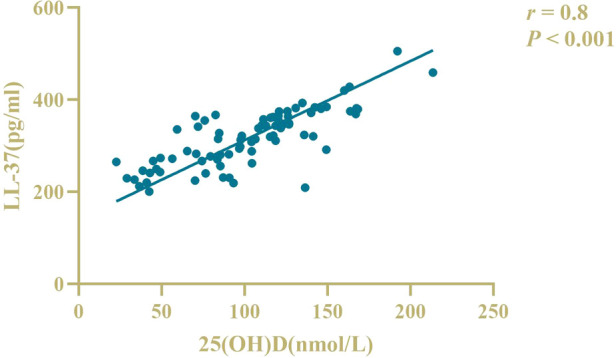
Correlation analysis of serum LL-37 and 25(OH)D levels in patients.

**Figure 3:**
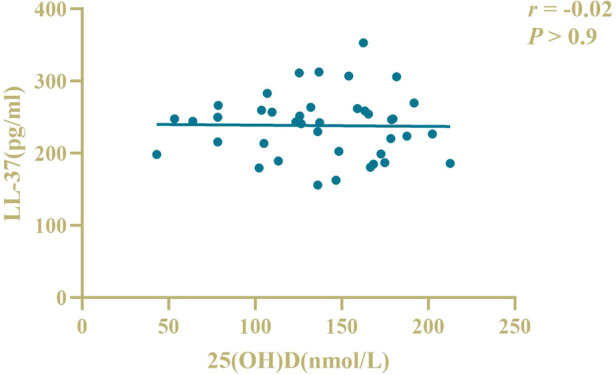
Correlation analysis of serum LL-37 and 25(OH)D levels in controls.

There was a positive correlation between serum 25(OH)D and LL-37 levels in patients (*r* = 0.8, *P *< 0.05), as shown in Figure 2. There was no correlation between serum 25(OH)D and LL-37 levels in controls (*r* = −0.02, *P* > 0.05), as shown in Figure 3.

#### Correlation analysis between the serum 25(OH)D level and clinical data in patients

(1) The serum 25(OH)D level was not correlated with the white blood cell count (WBC, *r_s_* = 0.003, *P* > 0.05) and C-reactive protein (CRP) level (*r_s_ *= 0.1, *P *> 0.05), and it was negatively correlated with the PCT level (*r_s_* = −0.3, *P* < 0.05) and the length of hospital stay (*r_s_* = −0.3, *P* < 0.05), as shown in Table 2.
(2) Serum LL-37 levels were not correlated with the WBC count (*r_s_* = 0.08, *P* > 0.05) and CRP levels (*r_s_* = 0.01, *P* > 0.05), and they were negatively correlated with PCT level (*r_s_* = −0.4, *P* < 0.05) and the length of hospital stay (*r_s_* = −0.2, *P *< 0.05), as shown in Table 2.

**Table 2:** Correlation of serum 25(OH)D and LL-37 with clinical data in patients.

	25 (OH)D (nmol/L)		LL-37 (pg/ml)	
	*r_s_* (95%CI)	*P*	*r_s_* (95%CI)	*P*
WBC (10^9^/L)	0.003	>0.9	0.08	0.4
CRP (mg/L)	0.1	0.3	0.01	0.9
PCT (ng/ml)	−0.3	0.004	−0.4	0.001
Days	−0.3	0.003	−0.2	0.04

## Discussion

Vitamin D deficiency is a risk factor for respiratory tract infection in children. There is a correlation between recurrent upper respiratory tract infection and vitamin D deficiency, and the lower the vitamin D level, the higher the frequency of upper respiratory tract infection (17). Vitamin D deficiency is present in patients with acute lower respiratory tract infection. There is an association between vitamin D deficiency and the severity of pneumonia. The severity of pneumonia in patients with vitamin D deficiency indicates that the serum vitamin D level can be used as a predictor of the severity and outcome of pneumonia (18). This study showed that the serum 25(OH)D level was decreased in the bacterial pneumonia group, the 25(OH)D level was lower in the severe pneumonia group than in the mild pneumonia group, and the vitamin D insufficiency rate was significantly higher in the severe pneumonia group than in the mild pneumonia group. Correlation analysis suggested that the serum 25(OH)D level was negatively correlated with the length of hospital stay and PCT, suggesting that vitamin D deficiency is a common problem in children with severe pneumonia and is related to the severity and outcome of pneumonia in infant.

LL-37 is the only antimicrobial peptide in the human body with an amphipathic *α*-helical structure that has a charge of +6 at neutral pH (19). It not only directly binds to the pathogens through the above positively charged structures and destroys bacterial membranes to cause direct sterilization (20), but also promotes the body to resist the invasion of pathogenic microorganisms through immune regulation and chemotaxis (21). Zhu C et al. (22) pointed out that the level of antimicrobial peptide LL-37 was higher in children with bacterial pneumonia than in normal healthy children in the study of children with pneumonia, and they speculated that LL-37 may be used as a biological marker in patients with bacterial pneumonia. Serum LL-37 was elevated *in vivo* in children with bacterial pneumonia in the study, which is consistent with the findings presented by Zhu C et al. Chen D et al. (23) demonstrated in their study that LL-37 levels were significantly higher in patients with severe pneumonia than in patients with mild pneumonia. However, the data obtained in this study showed that the serum LL-37 level was lower in children with severe pneumonia than in children with mild pneumonia, and the LL-37 level in children with pneumonia was negatively correlated with the PCT level and length of hospital stay, indicating that the LL-37 level decreased with aggravation of the disease, which was inconsistent with the study conclusion presented by Chen D et al., and the possible reasons were analyzed as follows: vitamin D could play a role in regulating LL-37 in the 50–125 nmol/L state (24), and the vitamin D level in some children with severe pneumonia was lower than 50 nmol/L and could not effectively produce LL-37.Therefore, whether LL-37 levels can be used as a marker to assess the severity of pneumonia remains to be further investigated in clinical studies. Whether there is a difference in the serum LL-37 level between children with pneumonia caused by different types of pathogens. Due to the very small sample size of some pathogens, relevant statistical analysis was not performed. In this study, only gram-positive bacteria and gram-negative bacteria were studied in groups, showing no difference in the serum LL-37 level between the two groups. A large sample size and multi-center study are needed for further discussion in the future.

Larcombe L et al. (25) suggested that there was no correlation between 25(OH)D levels and LL-37 levels in healthy children, while there was a positive correlation between 25(OH)D levels and LL-37 levels in children with pulmonary infection. Wang B et al. (26) found that 25(OH)D in the peripheral blood of children with recurrent upper respiratory tract infection was positively correlated with the antimicrobial peptide LL-37 content, and there was a direct relationship between LL-37 deficiency and vitamin D deficiency. The results showed that serum 25(OH)D levels were positively correlated with LL-37 levels in children. However, there was no correlation between serum 25(OH)D and LL-37 levels in healthy infants, and the reason for this occurrence may be that serum LL-37 levels in healthy infants reflect the systemic innate immune status of the body, while different levels of vitamin D immunoregulatory function cannot show significant differences at systemic concentrations. When the body enters a pathological state, under the induction of vitamin D, various types of immune cells and epithelial cells at related sites throughout the body begin to express a large amount of LL-37, thus, causing an increase in the serum LL-37 level and showing a correlation.

This study also has several limitations. First, this is a single center study, so the results may not be generalizable. Second, although we did 2 sputum cultures on all children to improve pathogenic accuracy, there may have been selection bias because sputum culture cannot be used as the gold standard for the pathogenic diagnosis of bacterial pneumonia and the control data selected for this study were from infants who underwent health checks at hospital outpatient departments.

In summary, low levels of vitamin D are present in infants with bacterial pneumonia, and their status affects the severity and outcome of pneumonia. LL-37 levels are increased in infants with bacterial pneumonia, but they show a decreasing trend with disease aggravation.

## Data Availability

The raw data supporting the conclusions of this article will be made available by the authors, without undue reservation.
